# Hate crimes against LGBT people: National Crime Victimization Survey, 2017-2019

**DOI:** 10.1371/journal.pone.0279363

**Published:** 2022-12-21

**Authors:** Andrew R. Flores, Rebecca L. Stotzer, Ilan H. Meyer, Lynn L. Langton

**Affiliations:** 1 Department of Government, School of Public Affairs, American University, Washington, District of Columbia, United States of America; 2 The Williams Institute, UCLA School of Law, Los Angeles, California, United States of America; 3 The Thompson School of Social Work and Public Health, University of Hawai’i, Mānoa, Honolulu, Hawai’i, United States of America; 4 RTI International, Washington, District of Columbia, United States of America; Oxford University Hospitals NHS Foundation Trust, UNITED KINGDOM

## Abstract

We estimate the prevalence and characteristics of violent hate crime victimization of lesbian, gay, bisexual, and transgender (LGBT) people in the United States, and we compare them to non-LGBT hate crime victims and to LGBT victims of violent non-hate crime. We analyze pooled 2017-2019 data from the National Crime Victimization Survey (*n* persons = 553, 925;*n* incidents = 32, 470), the first nationally representative and comprehensive survey on crime that allows identification of LGBT persons aged 16 or older. Descriptive and bivariate analysis show that LGBT people experienced 6.6 violent hate crime victimizations per 1,000 persons compared with non-LGBT people’s 0.6 per 1,000 persons (odds ratio = 8.30, 95% confidence interval = 1.94, 14.65). LGBT people were more likely to be hate crime victims of sexual orientation or gender bias crime and less likely to be victims of race or ethnicity bias crimes compared to non-LGBT hate crime victims. Compared to non-LGBT victims, LGBT victims of hate crime were more likely to be younger, have a relationship with their assailant, and have an assailant who is white. Compared to LGBT victims of non-hate violence, more LGBT hate crime victims reported experiencing problems in their social lives, negative emotional responses, and physical symptoms of distress. Our findings affirm claims that hate crimes have adverse physical and psychological effects on victims and highlight the need to ensure that LGBT persons who experience hate crime get necessary support and services in the aftermath of the crime.

## Introduction

Hate crime laws in the US often take two forms; one that outlines requirements for identifying and reporting hate crimes that occur in the jurisdiction, and one that details some type of additional punishment, often in the form of sentence enhancements, for people convicted of hate crimes. At the federal level, sexual orientation and gender identity (SOGI) have been protected categories for just over a decade, since the passage of the Matthew Shepard and James Byrd Jr., Hate Crimes Act of 2009. Places like California and Washington DC included SOGI as protected categories in their hate crime laws long before the federal government. However, of the 46 states and DC that have enacted state hate crime laws, 37 include sexual orientation as a protected category and 28 include gender or gender identity [1]. While inclusion of SOGI into hate crime laws was originally conceptualized as a victory for the lesbian, gay, bisexual, and transgender (LGBT) community and a recognition of their heightened risk for prejudice-motivated conduct [2, 3], more recent scholars have begun to question whether hate crime laws, particularly the use of sentence enhancements, offer protection for LGBT victims or create another means of policing vulnerable communities [4].

To understand the rates of hate crime among vulnerable community members and the harms that are associated with them, it is necessary to have systematic documentation and transparent reporting of these crimes. The hate crime laws that have mandated identification and tracking of these crimes have allowed for more detailed study of prejudice-motivated violence against LGBT communities than earlier work that utilized convenience samples alone. Prior scholars have noted the difficulty in tracking the prevalence and harms of prejudice-motivated violence due to a reliance on small, regionally-specific samples with limited generalizability, and the issues with official counts provided by law enforcement [5]. Research from small-scale, non-probability samples, or geographically limited studies suggest that LGBT people are at a heightened risk of experiencing hate crimes than the general population and that the mental health consequences of these experiences are pronounced.

In the first national probability sample of LGB people in the United States, Herek [6] found that roughly 20% of the sample had experienced a sexual orientation-motivated crime against their property or person since they had turned 18. Other studies suggested that gay and bisexual men bear a disproportionate burden of hate crime [6, 7], especially hate crimes “against persons,” meaning those that target people (such as assault or robberies) as opposed to crimes against property (such as arson or burglary) [8]. Some studies suggested that gay men and transgender women, particularly black, indigenous, people of color, were more likely to be murdered, although estimates of homicide risk vary greatly depending on the sample used [9–11]. Perpetrators of hate crimes against LGBT persons were disproportionately likely to offend in groups as opposed to alone and were most likely to be young men [12] who were strangers to the victim [13, 14]. Research also suggested that hate crimes against LGBT people disproportionately occurred in public spaces like streets, parking garages, or public transit, as opposed to private homes, workplaces or stores [13].

Studies showed that victims of hate crimes reported more adverse mental health (e.g., post-traumatic stress) [14–17] and physical health problems (e.g., difficulty sleeping and stomach ailments) [18] compared to victims of non-hate crimes. LGBT people with histories of experiencing discrimination and/or anti-LGBT violence were more at risk of suicidality and substance use issues [19, 20], and even being in communities that had higher incidences of LGBT hate crimes were linked to increased suicidality for LGBT youth in those communities [21]. However, these studies on the effects of hate crimes were based on convenience samples of LGBT people that were not generalizable to the larger population.

At the national level, the Federal Bureau of Investigation (FBI) collects data on hate crimes through the Uniform Crime Reports (UCR), and the National Crime Victimization Survey (NCVS) collects data on hate crime through the surveying of a representative sample of US households. The UCR only documents hate crimes that are reported to law enforcement agencies (LEAs), identified by LEAs as being hate-motivated, and then voluntarily reported by LEAs to the FBI. UCR hate crime data are also subject to errors due to nonresponse and data imputation processes [22]. The NCVS makes up for some of the UCR shortcomings by collecting self-reported data from US residents on their experiences with hate crimes both reported and not reported to police. Although these two data sources present the most comprehensive pictures of hate crime nationwide, including hate crimes motivated by SOGI bias, both historically have had limitations for understanding the frequency and nature of hate crime against LGBT persons.

Until recently the UCR and NCVS were also both limited in the information they could provide about hate crimes against LGBT people because both collections focused on the offenders’ motivation and did not collect data about the victims’ actual SOGI. In addition to being unable to compute a rate of hate crime victimization among LGBT persons, this method of reporting the data meant that current SOGI-motivated hate crime statistics include victimizations where the victim was cisgender and/or straight and left undocumented the reality that LGBT people might be victims of numerous types of bias-motivated violence (e.g., race-based violence). In other words, our national estimates and understanding of SOGI hate crimes did not fully describe the extent and nature of bias crimes against LGBT persons. This limitation in reporting was common for other bias-motivated categories as well (i.e., race bias incidents does not require that all victims be racial minorities), but the NCVS documented the race, ethnicity, and sex of victims, which could provide victimization rates and characteristics of hate crimes specific to the characteristics of victims. This had not been the case for SOGI.

With the limited research to date, the US lacks a clear picture of the extent and nature of hate crimes against LGBT persons, including the rate of hate crime among this population, the type of bias motivating the crime, and the distinct psychological and physical responses LGBT victims have to hate crime. In 2016, the NCVS began documenting the SOGI of respondents. However, to date, these data have not been explored for the purpose of understanding hate crimes against LGBT victims.

### Research hypotheses

This paper presents the first examination of the personal violent hate crime experiences of LGBT persons in a large probability sample of US residents. Given the prior literature, we expect that our examination of will support the following hypotheses:

*H*1. LGBT people have higher rates of hate crime victimization than non-LGBT people regardless of motivation.
*H*2. LGBT hate crime victims will differ from non-LGBT hate crime victims in the characteristics of victimization, such as the location of the hate crime victimization (e.g., public versus private spaces), victim-offender relationship (e.g., unknown versus known offenders), and offender characteristics (e.g., age of the offender).
*H*3. LGBT hate crime victims will report more adverse mental and physical health symptoms than LGBT non-hate crime victims.

We further seek to examine what hate crime victims report as the type of bias that motivated the victimization.

## Materials and methods

### Data

The NCVS is a stratified, multistage cluster sample with a rotating panel survey of households and persons. As a rotating panel, households are recruited and empaneled and others roll off on a continuous basis, and interviews are conducted throughout the year even though households are only interviewed every six months. The first interview takes into account lifetime victimizations such that subsequent interviews identify victimizations that occurred in the previous six months. The sampling frame includes all households including group quarters (e.g,. dormitories) but not military housing or institutional settings (e.g., correctional facilities). The United States is divided into 1,987 primary sample units (PSUs) based on population and geographic size that are then divided into sampling strata, and strata containing larger geographies are selected with certainty while remaining strata are sampled proportionate to their size. Selected households are empaneled for three years. All household members aged 12 years or older are interviewed every six months about their victimization experiences, resulting in over 200,000 interviews each year. Interviews are mostly conducted over the telephone with initial interviews primarily occurring in person by trained interviewers from the US Census Bureau. The response rates for the NCVS are typically over 80% at the person-level.

Two broad categories of crime are measured in the NCVS: personal or violent victimizations and property victimizations. Respondents are asked a series of screening questions to gauge whether they may have experienced a violent or property crime during the prior six months. If they answer any of the screener questions affirmatively, they are then asked a series of questions about the nature of the incident that are used to classify the type of crime experienced. *Violent victimizations* include simple assaults and serious violent crimes. Simple assault includes incidents involving an attack, attempted attack, or threat to attack a victim without a weapon and incidents that resulted in minor injury with less than 2 days of hospitalization. Serious violent crime includes incidents involving rape, sexual assault, robbery, aggravated assault, and a weapon and incidents that resulted in injury with 3 or more days of hospitalization. *Property victimizations* include burglary, motor vehicle theft, and other theft. Questions about violent victimizations are asked of each individual respondent while questions about property crime are asked only of one household respondent. Because the vast majority of NCVS hate crimes are violent crimes and the focus is on individual experiences with victimization, this study focuses solely on violent victimizations.

We aggregated NCVS data from 2017 to 2019, the years for which SOGI measures are included on available NCVS public-use data files at the National Archive of Criminal Justice Data [23]. All results reported here considered the complex design characteristics of the NCVS, including its rotating panel, when presenting point estimates, standard errors, and confidence intervals. This included the use of appropriate weights, and standard errors were estimated via direct variance estimation [24]. All analyses were performed in Stata SE v. 14.2. Greater description of the sampling and weighting of the NCVS data files can be found in the BJS reports (e.g., [25, p. 11–15]) and technical documentation [26].

### Measures

#### Sexual orientation and gender identity

Beginning in mid-2016, the Bureau of Justice Statistics (BJS) added SOGI questions to the NCVS for respondents aged 16 or older [27]. With the addition of these questions, the NCVS became the first household survey to collect nationally representative data on SOGI [27]. From 2016 to June 2019, all direct survey respondents aged 16 or older were asked about their sexual orientation, assigned sex at birth, and current gender identity. However, beginning in July 2019, BJS changed the questionnaire to ask only people aged 16 or older their SOGI if they reported a victimization, meaning that the survey data could no longer be used to generate a population estimate of LGBT persons, which was needed for calculating rates of crime against LGBT persons. For this reason, analyses that present summary data or victimization rates used NCVS data from January 2017 to June 2019, the period during which SOGI measures were collected from the full sample of respondents age 16 or older.

About 2% of the population 16 or older identified their sexual orientation as LGB, and 0.05% identified transgender as their current gender. Following established practices [23], we categorized people as LGBT if they identified as LGB or transgender, or if they had a current gender identity that was different from their assigned sex at birth. We categorized people as non-LGBT if they identified as straight and had a current gender identity that was the same as their assigned sex at birth. People who did not respond in a way that fits into one of these two categories (e.g., “something else”) were categorized separately. This categorization resulted in 2.1% of the sample being classified as LGBT (CI = 2.0, 2.2; n = 10,533), 94.9% as non-LGBT (CI = 94.6, 95.2; n = 527,846), and 3.1% as unknown (CI = 2.8, 3.3, n = 15,546).

#### Hate crime

All NCVS respondents who experienced victimization were asked detailed series of questions for each incident to classify the type of crime and gather additional details about their victimization. Each unique incident was classified separately, so a victim of multiple crimes would have documentation for each. Among other questions, victims were asked:

“Hate crimes or crimes of prejudice or bigotry occur when an offender/offenders target(s) people because of one or more of their characteristics or religious beliefs. Do you have any reason to suspect the incident just discussed was a hate crime or crime of prejudice or bigotry?”

If a victim suspected the incident was motivated by bias, the NCVS interviewer asked a series of follow-up questions to determine the perpetrator’s bias motivation and the reason why the victim believed it had been a hate crime. The Hate Crimes Statistics Act of 1990 defines a hate crime as “crimes that manifest evidence of prejudice based on race, religion, sexual orientation, or ethnicity” [28, p. 140]. Following the federal definition of hate crime, the types of bias asked about in the NCVS include protected categories of race, ethnicity, religion, disability, gender, and sexual orientation [29, p. 2]. Gender was not defined in this portion of questionnaire. The exact wording was, “An offender/offenders can target people for a variety of reasons, but we are only going to ask you about a few today. Do you suspect offender(s) targeted you because of your gender?” The NCVS also included two questions about whether the victim believed they were targeted because of the people they were associated with or because of the offender’s perception that they belonged to a protected category. Victims could select multiple types of bias-motivation from the listed characteristics for each incident they suspected they were victims of a hate crime. Since it was likely that hate crimes against LGBT victims may be motivated by sexual orientation or gender (e.g., [30]), we created a separate indicator if the victim said the bias was based on sexual orientation, gender, or both.

Not all incidents believed to be motivated by one of these types of bias qualify as hate crimes in official BJS counts. BJS only defined an NCVS incident as a hate crime if the victim had one of three types of evidence that the crime was motivated by bias: the offender used hate language, left hate symbols at the scene, or police investigators stated that the incident was a hate crime. For consistency, we largely followed the BJS definition of a hate crime and focused our analysis on victimizations motivated by bias that involved hate language, hate symbols, or some confirmation by police as evidence that the incident was a hate crime. However, in the Supporting Information section, we included analyses of all violent crimes suspected to be hate crimes.

#### Victimization characteristics

We examined the following characteristics of violent hate crimes: the demographic characteristics of victims, location of the incident, the relationship between the victim and the offender, the demographics of offenders, and whether victimizations resulted in problems at work/school or with family/friends.

#### Mental health correlates of victimization

Victims were asked how distressing the incident was on a four-point scale ranging from “Not at all distressing” to “Severely distressing.” We dichotomized this measure to summarize distress (1 = Moderately or severely distressing; 0 = Not at all or mildly distressing). Victims who reported feeling moderately or severely distressed, who said being a victim led to problems at work or school, or who said being a victim led to problems with family members or friends were also asked whether they had experienced specific mental health symptoms for a month or more following the victimization. These symptoms included feeling: worried or anxious; angry; sad or depressed; vulnerable; distrusting, or unsafe, and they are each coded 1 if a respondent answered affirmatively. Respondents who said they were not distressed, who said being a victim did not lead to problems at work or school or with family members or friends were coded 0 on the specific mental health symptoms. Victims were also asked if they sought professional help for the emotional distress they felt as a result of being a victim.

#### Physical health correlates of victimization

In addition to reporting physical injuries directly resulting from the victimization, victims who reported feeling moderately or severely distressed, had problems at work or school, or had problems with family members or friends were also asked about any physical health problems they had for a month or more following the victimization, including: headaches; trouble sleeping; changes in eating and drinking habits; upset stomach; high blood pressure; and muscle tension, and they are each coded 1 if a respondent answered affirmatively. Respondents who said they were not distressed, who said being a victim did not lead to problems at work or school or with family members or friends were coded 0 on the physical health problems. Victims were also asked if they sought professional help for the physical problems they experienced as a result of being a victim.

### Analysis

We present two primary estimates of victimization: victimization rates and victimization percentages. Victimization rate is the number of victimizations per 1,000 persons in the population. To address the first hypothesis, we compare rates of hate crime for LGBT and non-LGBT persons, using pooled NCVS data from January 2017 to June 2019. These rates take into account multiple victimizations against a single individual. The remaining analyses examine whether victimization experiences and correlates differ between LGBT and non-LGBT victims of violent hate crime or LGBT violent hate crime and non-hate crime victims. For these analyses, we use victimization percentages, which detail the characteristics of victimizations (e.g., the percent of violent victimizations that are hate crimes). Because victimization percentages are computed using SOGI measures collected from victims rather than the whole sample, these analyses use pooled NCVS data covering the full period January 2017 to December 2019. Victimization percentages are estimated at the incident-level, so a single victim will have multiple observations if there were multiple incidents. Consistent with BJS practices, we constrain our analyses to victimizations that occur within the United States. We conduct the analyses using weighted data, specifying the complex sample design strata and clusters. We report odds ratios with 95% CI. Odds ratios (*OR*s) comparing LGBT and non-LGBT people for estimated victimization rates $\left(\hat{VR}\right)$ are calculated by:

$$\begin{array}{c} OR=\frac{{\hat{VR}}_{LGBT}}{1000-{\hat{VR}}_{LGBT}}÷\frac{{\hat{VR}}_{Non-LGBT}}{1000-{\hat{VR}}_{Non-LGBT}}. \end{array}$$ (1)

*OR*s for victimization percentages or on the likelihood a person is a hate crime victim are from logistic regressions. We report estimates for both violent hate crime and violent non-hate crime.

## Results

### Rates and types of hate crime victimization by SOGI

The violent hate crime victimization rate for LGBT people was 6.6 (SE = 2.3) victimizations per 1,000 LGBT persons compared to 0.8 (SE = 0.1) victimizations per 1,000 non-LGBT persons (OR = 8.30, CI = 1.94, 14.65). The violent SOGI-based hate crime victimization rate for LGBT people was 5.4 (SE = 2.2) victimizations per 1,000 LGBT people compared to 0.2 (SE = 0.08) victimizations per 1,000 non-LGBT people (OR = 25.54, CI = 0.00, 53.47).

About 9.2% (SE = 2.6) of all violent victimizations against LGBT victims were hate crimes compared to 4.1% (SE = 0.5) of all violent victimizations with non-LGBT victims (OR = 2.39, CI = 1.19, 4.81). Relative to non-LGBT people, LGBT people had higher odds of being victims of a violent hate crime (OR = 8.99, CI = 5.70, 14.16).

Of violent hate crimes, 66.8% (SE = 9.9) against LGBT victims were simple assaults compared to 69.8% (SE = 5.2) against non-LGBT victims (OR = 0.87, CI = 0.30, 2.55). Among serious violent crimes, 22.9% (SE = 8.2) against LGBT victims were robberies compared to 5.2% (SE = 2.0) against non-LGBT victims (OR = 5.36, CI = 1.41, 20.41), and 4.5% (SE = 2.7) against LGBT victims were aggravated assaults compared to 21.9% (SE = 4.9) against non-LGBT victims (OR = 0.17, CI = 0.04, 0.69).

The types of bias that motivated violent hate crimes against LGBT and non-LGBT victims are documented in Table 1. Overall, 84.7% of LGBT hate crime victims compared to 25.0% of non-LGBT hate crime victims identified gender or sexuality as the bias motivation; 14.3% percent of LGBT hate crime victims identified biases other than gender or sexuality as the motivation for the hate crime. The results shown in Table 1 obscured patterns by race and gender. Large proportions of LGBT people of color reported bias motivation for race (40.4%, SE = 7.5), ethnicity (36.1%, SE = 14.1), and gender or sexuality (64.4%, SE = 14.8). A smaller proportion of GBT men report bias motivation for gender or sexuality (69.7%, SE = 14.6) than LBT women (94.4%, SE = 4.7).

**Table 1:** The type of bias-motivation among hate crime victims, by sexual orientation and gender identity, United States, National Crime Victimization Survey 2017–2019.

	LGBT		Non-LGBT		*OR*	(95%*CI*)
	%	(*SE*)	%	(*SE*)	(ref. Non-LGBT)	
Race	16.1	(6.7)	74.8	(4.9)	**0.06**	**(0.02, 0.20)**
Religion	10.4	(5.7)	12.6	(3.0)	0.81	(0.21, 3.09)
Ethnicity	11.2	(5.5)	40.0	(6.4)	**0.19**	**(0.06, 0.64)**
Disability	5.3	(4.1)	8.8	(2.7)	0.59	(0.11, 3.15)
Gender	38.9	(13.8)	23.6	(6.5)	2.05	(0.53, 7.98)
Sexuality	75.2	(9.1)	1.9	(0.9)	**156.4**	**(40.4, 606.2)**
Association^a^	19.7	(7.8)	29.0	(6.7)	0.60	(0.19, 1.87)
Perceived characteristics^b^	4.8	(2.3)	9.4	(2.4)	0.48	(0.15, 1.55)
Gender or sexuality (combined)	84.7	(7.0)	25.0	(6.5)	**16.5**	**(4.60, 59.6)**

*Note*: Victims could select more than one bias motivation that apply to their victimization. Bold odds ratios and confidence intervals are statistically significant at *p* < .05.

^a^ Association refers to the victim’s perception of victimization was motivated by the victim’s association with people who have certain characteristics or religious beliefs.

^b^ Perceived characteristics refers to the victim’s perception of victimization was motivated by the offender’s perception of the victim’s characteristics or religious beliefs even if inaccurate.

### Characteristics of violent hate crime victimization

Table 2 documents whether LGBT and non-LGBT hate crime victims differ in their characteristics, the location of the hate crime victimization, victim-offender relationship, and offender characteristics. Compared to non-LGBT victims, LGBT victims were more often below age 35 (OR = 4.4, CI = 1.6, 12.3), well-known to their assailant (OR = 8.2, CI = 2.0, 33.7), with a White offender (OR = 5.9, CI = 1.6, 21.9), and less often a stranger to the assailant (OR = 0.26, CI = 0.08, 0.81). The large confidence intervals reflected the small number of LGBT victims, but other apparent differences were noteworthy though with greater uncertainty, including that LGBT victims were more likely than non-LGBT victims to be women, that LGBT hate crime incidents more frequently occurred near the victims’ homes, and that LGBT hate crime incidents were more often in suburban areas compared with incidents of non-LGBT victims.

**Table 2:** Victimization characteristics of violent hate crime, LGBT versus non-LGBT, United States, National Crime Victimization Survey 2017–2019.

	LGBT		Non-LGBT		Difference		*OR*	(95%*CI*)
	%	(*SE*)	%	(*SE*)	%	(*SE*)	(ref. Non-LGBT)	
**Victim Characteristics**								
*Current Gender*								
Man	29.3	(10.3)	54.1	(6.5)	**-24.8**	**(12.3)**	0.35	(0.11, 1.1)
Woman	61.2	(12.4)	45.9	(6.5)	15.2	(14.0)	1.85	(0.58, 5.9)
Non-binary	3.9	(2.9)	–	–	–	–	–	–
Person of color	29.1	(10.5)	43.7	(6.6)	-14.6	(12.0)	0.53	(0.17, 1.6)
Below 35 years old	73.2	(9.7)	38.2	(5.9)	**34.9**	**(10.5)**	**4.4**	**(1.6, 12.3)**
Annual income < $25,000	29.8	(10.6)	30.9	(5.4)	-1.1	(12.0)	0.95	(0.31, 2.9)
**Location of Incident**								
At or near victim’s home	54.6	(13.9)	30.4	(6.2)	24.2	(15.3)	2.8	(0.78, 9.7)
In public space	22.8	(8.9)	34.2	(5.2)	-11.4	(10.3)	0.57	(0.19, 1.7)
**Victim-Offender Relationship**								
Well-known	49.1	(14.9)	10.7	(3.7)	**38.4**	**(15.4)**	**8.1**	**(2.0, 33.2)**
Stranger	36.7	(11.9)	70.2	(4.4)	**-32.4**	**(13.2)**	**0.25**	**(0.08, 0.78)**
**Urbanicity of Victim’s Residence**								
Urban	26.1	(9.1)	46.2	(5.9)	-20.1	(10.4)	0.41	(0.15, 1.13)
Suburban	65.9	(11.2)	47.3	(6.0)	18.7	(12.6)	2.2	(0.73, 6.4)
Rural	8.0	(5.5)	6.5	(2.7)	1.5	(6.0)	1.2	(0.23, 6.7)
**Offender Characteristics**								
*Offender Sex*								
Male(s)	74.3	(17.8)	70.0	(5.9)	4.4	(18.5)	1.2	(0.18, 8.4)
Female(s)	24.0	(18.0)	20.1	(4.6)	3.9	(18.5)	1.3	(0.17, 9.52)
Male(s) and female(s)	1.6	(1.7)	9.9	(4.6)	-8.3	(4.9)	0.15	(0.01, 1.5)
One offender	85.6	(6.7)	77.4	(4.7)	8.3	(8.7)	1.7	(0.49, 6.2)
White	87.5	(6.3)	54.4	(7.6)	**33.1**	**(10.0)**	**5.9**	**(1.6, 21.9)**
30 years old or older	62.5	(15.4)	60.2	(6.5)	2.3	(17.2)	1.1	(0.26, 4.7)

*Note*: None of the percentages should sum to 100 except for urbanicity of incident due to excluded categories that are not reported. Bold differences and odds ratios are statistically significant at *p* < .05.

### Social, mental, and physical health correlates of hate crimes among LGBT victims

Table 3 compares social, mental, and physical health correlates of violent victimizations among LGBT victims of hate crimes and LGBT non-hate crime victims.

**Table 3:** Effects of victimization of violent hate and non-hate crime among LGBT victims, United States, National Crime Victimization Survey 2017–2019.

	Violent Hate Victims		Violent Non-Hate Victims		Difference		*OR*	(95%*CI*)
	%	(*SE*)	%	(*SE*)	%	(*SE*)	(ref. Violent non-hate victims)	
**Problems in Social Life**								
Problems in work or school	31.5	(13.4)	23.3	(4.7)	8.2	(14.1)	1.5	(0.40, 5.7)
Problems with family or friends	58.5	(13.0)	21.4	(4.2)	**37.2**	**(13.4)**	**5.2**	**(1.7, 16.2)**
Sought help from victim agencies other than the police	9.3	(4.8)	11.7	(3.2)	-2.5	(5.3)	0.77	(0.23, 2.5)
**Emotional Responses to Victimization**								
Moderately or severely distressing to be a victim	61.2	(13.7)	50.7	(7.5)	10.5	(15.5)	1.5	(0.42, 5.5)
Worried or anxious	74.1	(9.5)	41.4	(6.2)	**32.6**	**(11.2)**	**4.0**	**(1.4, 12.1)**
Angry	78.3	(8.5)	42.1	(6.3)	**36.3**	**(10.6)**	**5.0**	**(1.6, 15.3)**
Sad or depressed	63.0	(12.1)	30.7	(5.2)	**32.2**	**(12.9)**	**3.8**	**(1.3, 11.6)**
Vulnerable	72.5	(9.8)	37.4	(6.1)	**35.1**	**(11.5)**	**4.4**	**(1.5, 13.2)**
Violated	74.1	(9.5)	34.3	(5.6)	**39.8**	**(11.3)**	**5.5**	**(1.8, 16.8)**
Mistrust	65.0	(11.5)	36.3	(6.5)	**28.6**	**(13.5)**	**3.2**	**(1.0, 10.5)**
Unsafe	75.2	(9.2)	39.0	(6.1)	**36.2**	**(11.2)**	**4.7**	**(1.6, 14.4)**
Sought professional help for emotional problems relating to victimization	39.3	(13.8)	18.3	(4.0)	21.0	(14.4)	2.9	(0.82, 10.2)
**Physical Responses to Victimization**								
Victimization resulting in injury	32.1	(13.4)	23.8	(4.3)	8.3	(14.4)	1.5	(0.40, 5.8)
Headaches	53.8	(14.0)	18.6	(4.9)	**35.2**	**(14.3)**	**5.1**	**(1.5, 17.3)**
Trouble sleeping	59.5	(12.9)	30.8	(5.3)	**28.7**	**(13.5)**	**3.3**	**(1.1, 10.2)**
Changes in eating or drinking	59.5	(13.0)	22.7	(4.8)	**36.4**	**(13.4)**	**4.9**	**(1.6, 15.4)**
Upset stomach	52.4	(14.4)	22.2	(4.8)	**30.2**	**(14.6)**	**3.9**	**(1.2, 12.9)**
Fatigue	58.4	(13.1)	27.5	(4.6)	**30.9**	**(13.8)**	**3.7**	**(1.2, 11.7)**
High blood pressure	35.1	(16.5)	8.1	(2.8)	27.0	(16.3)	**6.1**	**(1.4, 27.3)**
Muscle tension	55.2	(13.7)	22.5	(5.2)	**32.7**	**(14.2)**	**4.2**	**(1.3, 14.1)**
Sought professional help for physical problems relating to victimization	34.6	(16.4)	10.5	(3.2)	24.1	(16.8)	4.5	(0.91, 22.1)

*Note*: Bold differences and odds ratios are statistically significant at *p* < .05.

#### Problems in social life

Victims were asked whether they encountered any problems at work or school or with family or friends as a result of the victimization. The percentage of victimizations resulting in problems with work or school or family and friends was higher among LGBT victims of hate crimes than LGBT non-hate crime victims, though was only statistically different for work and school problems.

Overall, only about one in ten LGBT victims sought help from victim service agencies (other than police), which did not differ between hate crime and non-hate crime victims (9% vs. 12%, respectively).

#### Mental health correlates of hate crime victimization

About 61% of hate crime victims compared to 51% of non-hate crime victims reported feeling moderate to severe distress due to the victimization but this difference was not statistically significant. Despite small differences in a general measure of distress, LGBT victims of hate crime tended to report more often experiencing specific mental health responses than LGBT non-hate crime victims. For instance, more LGBT victims of hate crime than LGBT non-hate crime victims reported feelings of worry or anxiety, anger, sadness or depression, vulnerability, being violated, and feeling unsafe. About 39% of LGBT victims of hate crimes compared to 18% of LGBT victims of non-hate crimes reported seeking professional help for the emotional problems they experienced related to their victimization, though this difference was not statistically different.

#### Physical health correlates of hate crime victimization

There were no statistical differences in the extent to which LGBT violent hate crime and non-hate crime victims experienced physical injury directly resulting from the victimization (32% vs. 23%, respectively). However, more LGBT victims of hate crime than LGBT victims of non-hate crimes reported experiencing headaches, trouble sleeping, change in eating or drinking, upset stomach, fatigue, high blood pressure, and muscle tension. More than one-third (35%) of LGBT hate crime victims compared to 11% of LGBT non-hate crime victims sought professional help for the physical problems they experienced, but this difference was not statistically different.

## Discussion

Our findings largely support our research hypotheses. LGBT people have higher rates of hate crime victimization than non-LGBT people (*H*1), particularly hate crime motivated by anti-SOGI bias. The results show that LGBT people face many varied types of hate violence. We further find that a greater percentage of LGBT victims of hate-motivated violence to say that the bias-motivation was sexuality or gender compared to non-LGBT victims of hate-motivated violence.

Comparisons between LGBT and non-LGBT victims of violent hate crimes show interesting patterns of similarity and difference, which in part support *H*2. Thus, LGBT hate crime victims are more likely than non-LGBT hate crime victims to be younger. Neither the location of the crime (e.g., proximity to victim’s home) nor the urbanicity of the victim’s residence show statistical differences between LGBT and non-LGBT hate crime victims. In terms of offender characteristics, LGBT victims of hate-motivated violence are more likely than non-LGBT victims of hate-motivated violence to report that their perpetrator is White. In contrast to prior research findings [13, 14], a greater proportion of LGBT victims of hate-motivated violence compared to non-LGBT victims of hate-motivated violence report that the offender was known to them rather than strangers. The difference from prior research findings may be related to the generalizability of the samples in the prior studies and may be further exacerbated with how intra-familial and partner crimes are classified in research. In addition, official police reports may underrepresent some crimes that do not have overt stereotypical characteristics of hate crimes [31]. For example, incidences that happen among youth are often labeled “bullying” even when they may meet the definition of hate crime as well [32]. Thus, domestic violence, child abuse, and other types of crime that do not meet the stereotypical characteristics of hate crimes may be missed in police reports and are better represented in our report from the NCVS. This is plausible because prejudice against LGBT people often originates in their families and among people in their social networks, as family and acquaintances may take on monitoring the gender and sexuality of LGBT people. Further, this finding may also make intuitive sense, since sexual orientation and gender identity are considered concealable stigmas. Known others are more likely than strangers to know someone’s sexual orientation or gender identity, thus increasing the risk of homophobic or transphobic violence by known others. Further research on LGBT hate crime victims should investigate *known* versus *perceived* victim’s SOGI status and the relationship between the victim and the offender.

While there are no marked differences between LGBT victims of hate and non-hate crime in overall experiences of distress following a victimization, LGBT people experience more negative mental and physical health symptoms following a violent hate crime than other crimes of violence (*H*3). We find that most psychological and physical symptoms are more pronounced among LGBT victims of violent hate crime than LGBT non-hate crime victims. This may be related to patterns that Herek et al. [13] describe, where gay and lesbian people who attribute negative experiences to sexual prejudice are more psychologically distressed than those who do not make such attributions.

In addition, the findings suggest less than half of LGBT victims of violent hate crime sought out help from medical or mental health providers for those psychological or physical symptoms. Policy and legal discussions about the inclusion of sexual orientation and gender identity protections in hate crime legislation emphasize the adverse physical and psychological effects on victims [3, 4]. Our findings support those claims.

### Limitations

The analyses and findings presented here have several limitations that should be addressed in future research and as additional years of NCVS SOGI data are made available. The first is the small sample size of LGBT hate crime victims. These small sample sizes impact the precision of the estimates and make it difficult to assess differences between LGBT and non-LGBT victims and between LGBT hate crime and non-hate crime victims. The small sample sizes also limit the ability to assess the intersections of LGBT and other victim demographics like race and ethnicity and limit the ability to apply statistical controls. Future research when additional years of NCVS data are available should explore the intersections of SOGI and other identities in the context of hate crime victimization. In addition, confounders can be conditioned on to account for potential spurious relationships.

Another concern is in the limited range of NCVS variables and the lack of longitudinal data to address health outcomes related to hate crime victimization. Because the NCVS uses a 6-month reference period, some of the longer-term potential consequences of victimization in general, such as increased likelihood of substance use [33], posttraumatic stress disorder [34], and suicide risk [35], are not asked about in the survey. Future research should consider approaches to better understand the longer-term consequences of hate crime victimization among LGBT populations.

We focus our analysis on personal violent hate crimes because that is the most prevalent type of hate crime in the NCVS. Property hate crimes are also documented in the NCVS, but such incidents are less common. This may be an artifact of the way the NCVS documents property crime and bias-motivated crime. Due to small samples, we are unable to further examine property hate crimes for LGBT people.

Another limitation is that the NCVS relies on self-reports provided in an interview context. While U.S. Census Bureau interviewers are trained to obtain accurate data, there may be LGBT participants who did not disclose their sexual orientation, assigned sex at birth, or current gender identity. NCVS administrators have evaluated this before implementing the SOGI questions, but it may still limit the population of LGBT people to whom our findings generalize [27]. The sampling frame also misses individuals who lack stable housing or are in institutional contexts, where outcomes may be worse for the some of the most vulnerable in society.

There may be further limitations in requiring victims to identify whether their victimization was bias-motivated and their perception of the offender’s motivation. Victims may not know or misperceive their victimization. Indeed, BJS has funded external evaluations to enhance and improve the measurement of hate crimes in the NCVS [36]. How the NCVS defines and categorizes violence and hate-motivated violence may also differ from how LGBT community members may define and measure violence. This also includes community-level effects of individual victimization.

Finally, the changes to how the NCVS documents sexual orientation and gender identity may limit future analyses. Further work replicating what we report here will find complications in producing victimization rates. Analysts at BJS have had to construct different analytical approaches to the NCVS to produce a recent report of patterns of violence by sexual orientation and gender identity [37]. Future research will have to continue to devise such workarounds. NCVS administrators restored documenting sexual orientation and gender identity to all respondents aged 16 or over in 2022.

## Public policy and broader implications

Recent research has found that LGBT people, particularly transgender persons and bisexual women, are more likely to be victims of personal and property crimes [23, 38, 39]. Our findings demonstrate that some of these victimization experiences are rooted in bias-motivated hate, which heightens the harms and consequences for both direct victims and their community. These findings highlight the importance of developing and continuing to develop federal, state, and local interventions to protect LGBT people from victimization and provide support and services to mitigate the myriad adverse consequences of these victimization experiences.

However, prior research documents that law enforcement and antiviolence programs and services may be unprepared to effectively serve LGBT victims and address the full scope of their needs [40, 41]. In addition, the extant research on bias crime victim responses have demonstrated significant gaps and inadequate services that can further victimize people a they seek assistance [42], and very few countries offer trained specialist support for the unique needs of victims of hate crimes [43]. This may be due in part to a lack of understanding of the nature and effects of victimization among LGBT populations. For instance, our findings show that LGBT hate crime victims are more likely to be younger than other victims and be victimized by someone known to them, and about half of violent hate crime victimizations with a LGBT victim occur in or near their home. Additionally, our findings distinguish the unique mental and physical harm related to hate-motivated violence on LGBT victims, and suggest that among LGBT people, the harms of violent hate crime are greater and more severe than harms from non-hate crime violence.

Even though American’s attitudes have become more accepting of LGBT people and LGBT rights [44, 45], there has been a recent wave of anti-transgender and anti-LGB legislation in state legislatures [46]. Following Florida’s passage of Parental Rights in Education or a “Don’t Say Gay or Trans” law, there was a 406% increase in anti-LGBTQ rhetoric on social media tied to “groomer” language [47]. Protesters have also shut down libraries [48] and drag queen story hours [49] for providing affirming LGBTQ+ information to youth. The rise of extreme rhetoric and behaviors has the potential to embolden individuals to enact bias-motivated crimes against LGBT people. For example, during the contentious 2016 presidential elections that raised anti-LGBT rhetoric, transgender and gender non-conforming people reported increased exposure to hate speech and violence [50]. LGBT advocates further suggest that this rhetoric has direct connections to the Club Q mass shooting that occurred in late November 2022 [51]. Thus, our findings emphasize the importance of continued documentation of the victimization experiences of LGBT people.

One controversial intervention is enhanced punishments in hate crime laws, which add penalties to crimes motivated by hate. Scholars and community members have critiqued hate crime laws as supporting a carceral state and reinforcing narratives that harm LGBT communities. For example, scholars critique the justification for hate crime laws that have excess focus on the “stranger danger” argument that was used as part of the “tough on crime” approach or use examples of middle class gay white men as victims when hate crime victims are more diverse [52]. Our data do not directly speak to these questions, but they can advance the debate by providing some relevant data. For example, our findings demonstrate the complexity of victim identities, perpetrator characteristics, and the harms of bias-motivated violence. The results challenge the simplistic “stranger danger” narrative of hate crimes against LGBT persons.

Our findings demonstrate the need for the NCVS and other federal data collection efforts to continue to expand the extent to which SOGI demographic information are collected so we better understand the characteristics and harms of bias-motivated violence, discrimination, and criminal victimization risk and perpetration.

## Supporting information

S1 File Results for suspected hate crimes. (PDF)

## References

[1] Movement Advancement Project. Equality maps: Hate Crime Laws. Accessed June 23, 2021 from https://www.lgbtmap.org/equality-maps/hate_crime_laws.

[2] Jenness V, Brood K. Hate crimes: New social movements and the politics of violence. New York: Routledge; 1997.

[3] Jenness V, Grattet R. Making hate a crime: From social movement to law enforcement New York: Russell Sage Foundation; 2001.

[4] Woods J, Herman J. Anti-transgender hate crime. In: Hall N, Corbb A, Gianassi P, Greeves JGD, editors. The Routledge handbook on hate crime. New York: Routledge; 2014 pp. 278–288.

[5] Herek GM. Documenting hate crimes in the United States: Some considerations on data sources. Psychol. Sex. Orientat. Gend. Divers. 2017;42(2):143–151. doi: 10.1037/sgd0000227

[6] Herek GM. Hate crimes and stigma-related experiences among sexual minority adults in the United States: prevalence estimates from a national probability sample. J. Interpers. Viol. 2009;24(1):54–74. doi: 10.1177/0886260508316477 18391058

[7] Rothman EF, Exner D, Baughman A. The prevalence of sexual assault against people who identify as gay, lesbian, or bisexual in the United States: a systematic review. Trauma Violence Abuse. 2011;12(2):55–66. doi: 10.1177/1524838010390707 21247983 PMC3118668

[8] Stotzer RL. Comparison of hate crime rates across protected and unprotected groups—an update. Los Angeles: The Williams Institute.

[9] Boyler TL, Youk AO, Haas AP, Brown GR, Shipherd JC, Kauth MR, et al. Suicide, homicide, and all-cause mortality among transgender and cisgender patients in the Veterans Health Administration. LGBT Health 2021;8(3):173–180. doi: 10.1089/lgbt.2020.0235 33544021

[10] Dinno A. Homicide rates of transgender individuals in the United States: 2010–2014. Am. J. Pub. Health 2017;107(9):1441–1447. doi: 10.2105/AJPH.2017.303878 28727530 PMC5551594

[11] Gruenewald J, Kelley K. Exploring anti-LGBT homicide by mode of victims selection. Crim. Justice. Behav. 2014;41:1130–1160. doi: 10.1177/0093854814541259

[12] Coston L. Understanding characteristics of victims and perpetrators of anti-LGBT hate crimes in the United States Viol. Victims. 2018;33(3):453–471. doi: 10.1891/0886-6708.v33.i3.453 30567858

[13] Herek GM, Cogan JC, Gillis JR. Victim experiences in hate crimes based on sexual orientation. J. Soc. Issues. 2002;58(2):319–339. doi: 10.1111/1540-4560.00263

[14] Rose SM, Mechanic MB. Psychological distress, crime features, and help-seeking behaivors related to homophobic bias incidents. Am Behav Sci 2002;46(1):14–26. doi: 10.1177/0002764202046001003

[15] Herek GM, Gillis JR, Cogan JC, Glunt EK. Hate crime victimization among lesbian, gay, and bisexual adults. J. Interpers. Viol. 1997;12(2):195–215. doi: 10.1177/088626097012002003

[16] Herek GM, Gillis JR, Cogan JC. Psychological sequalae of hate crime victimization among lesbian, gay, and bisexual adults. J. Consult. Clin. Psychol. 1999;67(6):945–951. doi: 10.1037/0022-006X.67.6.945 10596515

[17] Clements-Noelle K, Marx R, Katz M. Attempted suicide among transgender persons: the influence of gender-based discrimination and victimization. J. Homosex. 2006;51(3):53–69. doi: 10.1300/J082v51n03_04 17135115

[18] Stotzer RL. Bias crimes based on sexual orientation and gender identity: global prevalence, impacts, and causes. In: Peterson D, Panfil VR, editors. Handbook on LGBT communities, crime, and justice. New York: Springer; 2014. pp. 45–64.

[19] McCabe SE, Bostwick WB, Hughes TL, West BT, Boyd CJ. The relationship between discrimination and substance use disorders among lesbian, gay, and bisexual adults in the United States. Am. J. Pub. Health 2010;100(10):1946–1952. doi: 10.2105/AJPH.2009.163147 20075317 PMC2937001

[20] Descamps MJ, Rothblum E, Bradford J, Ryan C. Mental health impact of child sexual abuse, rape, intimate partner violence, and hate crimes and the National Lesbian Health Survey. J. Gay Lesbian Soc. Serv. 2000;11(1):27–55. doi: 10.1300/J041v11n01_02

[21] Duncan DT, Hatzenbuehler ML. Lesbian, gay, bisexual, and transgender hate crimes and suicidality among a population-based sample of sexual-minority adolescents in Boston. Am. J. Pub. Health 2014;104(2):272–278. doi: 10.2105/AJPH.2013.301424 24328619 PMC3935714

[22] Maltz MD. Can we trust the FBI’s crime estimation procedures? Criminol. 2019;44(3):6–8.

[23] Flores AR, Langton L, Meyer IH, Romero AP. Victimization rates and traits of sexual and gender minorities in the United States: Results from the National Crime Victimization Survey, 2017. Sci. Adv. 2020;6:eaba6910. doi: 10.1126/sciadv.aba6910 33008905 PMC7852385

[24] Shook-Sa B, Couzens GL, Berzofsky M. User’s guide to the National Crime Victimization Survey (NCVS) direct variance estimation. Research Triangle Park: RTI international. 2011. [Cited 2021 Mar. 24]. Available from: https://www.bjs.gov/content/pub/pdf/NCVS_Variance_User_Guide%2011.06.14.pdf

[25] Morgan RE, Thompson A. Criminal victimization, 2020 Washington, DC: Bureau of Justice Statistics, 2020 Oct.;NCJ 301775. https://bjs.ojp.gov/sites/g/files/xyckuh236/files/media/document/cv20.pdf

[26] National Crime Victimization Survey, 2016: Technical documentation Washington, DC: Bureau of Justice Statistics, 2021 Dec.;NCJ 251442. https://bjs.ojp.gov/sites/g/files/xyckuh236/files/media/document/ncvstd16.pdf

[27] Truman JL, Morgan RE, Gilbert T, Vaghela P. Measuring sexual orientation and gender identity in the National Crime Victimization Survey. J. Off. Stat. 2019;35(4):835–858. doi: 10.2478/jos-2019-0035

[28] Hate Crimes Statistics Act of 1990, Pub. L. No. 101-275, 104 Stat. 140 (April 23, 1990).

[29] Kena G, Thompson A. Hate crime victimization, 2005–2019. Washington, DC: Bureau of Justice Statistics, 2021 Sep.;NCJ 300954. https://bjs.ojp.gov/sites/g/files/xyckuh236/files/media/document/hcv0519_1.pdf.

[30] Gordon AR, Meyer IH. Gender nonconformity as a target of prejudice, discrimination, and violence against LGB individuals. J. LGBT Health Res. 2007;3(3):55–71. doi: 10.1080/15574090802093562 19042905 PMC10790306

[31] Lantz B, Gladfelter AS, Ruback RB. Stereotypical hate crimes and criminal justice processing: A multi-dataset comparison of bias crime arrest patterns by offender and victim race. Justice Q. 2019;36(2):193–224. doi: 10.1080/07418825.2017.1399211

[32] Hall N, Hayden C. Is “hate crime” a relevant and useful way of conceptualizing some forms of school bullying? Int. J. Violence Sch. 2007 Apr:3–24.

[33] Logan TK, Walker R, Cole J, Leukefeld C. Victimization and substance abuse among women: contributing factors, interventions, and implications. Rev. Gen. Psychol. 2002;6(4):325–397. doi: 10.1037/1089-2680.6.4.325

[34] Kunst M, Winkel FW, Bogaerts S. Prevalence and predictors of posttraumatic stress disorder among victims of violence applying for state compensation. J. Interpers. Viol. 2010;25(2):1631–1654. doi: 10.1177/0886260509354591 20501900

[35] Barboza GE, Dominguez S, Chace E. Physical victimization, gender identity and suicide risk among transgender men and women. Prev. Med. Rep. 2016;4:385–390. doi: 10.1016/j.pmedr.2016.08.003 27547721 PMC4986045

[36] Langton L, Cook S, Krebs C, Hsieh YP, Timbrook J. Enhancing the measurement of hate crimes in the NCVS: Developing and testing improvements to the survey questions Research Triangle Park, NC: RTI International, 2021 Sep.;NCJ 301033. https://www.ojp.gov/pdffiles1/bjs/grants/301033.pdf

[37] Truman JL, Morgan RE. Violent victimization by sexual orientation and gender identity, 2017–2020. Washington, DC: Bureau of Justice Statistics, 2022 Jun.;NCJ 304277. https://bjs.ojp.gov/content/pub/pdf/vvsogi1720.pdf.

[38] Bender AK, Lauritsen JL. Violent victimization among lesbian, gay, and bisexual populations in the United States: findings from the National Crime Victimization Survey, 2017–2018. Am. J. Pub. Health 2021;111(2):318–326. doi: 10.2105/AJPH.2020.306017 33351656 PMC7811079

[39] Flores AR, Meyer IH, Langton L, Herman J. Gender identity disparities in criminal victimization: National Crime Victimization Survey, 2017–2018. Am. J. Pub. Health 2021;111(4):726–729. doi: 10.2105/AJPH.2020.306099 33600251 PMC7958056

[40] Brown TNT, Herman JL, Intimate partner violence and sexual abuse among LGBT people: a review of existing literature. Los Angeles: The Williams Institute. Retrieved June 23, 2021 from https://williamsinstitute.law.ucla.edu/wp-content/uploads/IPV-Sexual-Abuse-Among-LGBT-Nov-2015.pdf.

[41] Goldberg NG, Mallory C, Stemple L, Meyer IH. Police and the criminalization of LGBT people. In: Miller E, Lave T., editors. Cambridge handbook on policing in the United States. New York: Cambridge University Press; 2019. pp. 374–391.

[42] Chakraborti C. Responding to hate crime: Escalating problems, continued failings. Criminol. Crim. Justice 2018;18(4):387–404. doi: 10.1177/1748895817736096

[43] Enhancing Stakeholder Awareness and Resources for Hate Crime Victim Support. Hate crime victim support. Organization for Security and Co-operation in Europe Office for Democratic Institutions and Human Rights and the Association of Counseling Centers for Victims of Right-Wing, Racist, and Antisemitic Violence in Germany. 2020 [Cited 2022 Sep 7]. Available from: https://tandis.odihr.pl/bitstream/20.500.12389/23014/2/23014_EN.pdf

[44] Patterson CJ, Sepúlveda M, White J, editors. Understanding the well-being of LGBTQI+ populations. Washington, DC: The National Academies Press; 2020. 33104312

[45] Lewis DC, Flores AR, Haider-Markel DP, Miller PR, Taylor JK. Transitioning opinion? Assessing the dynamics of public attitudes toward transgender rights. Public Opin. Q. 2022;86(2):343–368. doi: 10.1093/poq/nfac014

[46] Lavietes M, Ramos E. Nearly 240 anti-LGBTQ bills filed in 2022 so far, most of them targeting trans people. NBC News. 2022 Mar 20 [Cited 2022 Aug 24]. Available from: https://www.nbcnews.com/nbc-out/out-politics-and-policy/nearly-240-anti-lgbtq-bills-filed-2022-far-targeting-trans-people-rcna20418

[47] Center for Countering Digital Hate and the Human Rights Campaign. Digital hate: Social media’s role in amplifying dangerous lies about LGBTQ+ people. Center for Counting Digital Hate, Inc. 2022 Aug 10 [Cited 2022 Aug 24]. Available from: https://hrc-prod-requests.s3-us-west-2.amazonaws.com/CCDH-HRC-Digital-Hate-Report-2022-single-pages.pdf

[48] Paquette D. A Mich. library refused to remove an LGBTQ book. The town defunded it. The Washington Post. 2022 Aug 24 [Cited 2022 Aug 24]. Available from: https://www.washingtonpost.com/nation/2022/08/24/michigan-library-defunded-gender-queer/

[49] Bikales J. Drag exploded in popularity. Then came the protests and attacks. The Washington Post. 2022 Aug 12 [Cited 2022 Aug 24]. Available from: https://www.washingtonpost.com/nation/2022/08/12/drag-mainstream-attacks-crossroads/

[50] Veldhuis CB, Drabble L, Riggle EDB, Wootton AR, Hughes TL. “I fear for my safety, but want to show bravery for others”: Violence and discrimination concerns among transgender and gender non-conforming individuals after the 2016 presidential election. Violence Gend. 2018;5(1):26–36. doi: 10.1089/vio.2017.0032

[51] Hernandez E. Anti-LGBTQ rhetoric leads to violence, advocates say in wake of Club Q shooting. The Denver Post. 2022 Nov 20 [Cited 2022 Nov 28]. Available from: https://www.denverpost.com/2022/11/20/club-q-shooting-anti-lgbtq-rhetoric-violence-colorado/

[52] Meyer D. Resisting hate crime discourse: Queer and intersectional challenges to neoliberal hate crime laws. Crit. Criminol. 2014;22(1):113–125. doi: 10.1007/s10612-013-9228-x

