# Supplementary material for: Hate crimes against LGBT people: National Crime Victimization Survey, 2017-2019
Source: PLoS One. 2022 Dec 21;17(12):e0279363. doi: 10.1371/journal.pone.0279363 (PMC9770371; doi:10.1371/journal.pone.0279363)
Supplement: S1 File — (PDF) [file pone.0279363.s001.pdf]

S1 Supporting information

In the main text, we report results based on the way the Bureau of Justice Statistics classifies a victimization as a hate crime. Not only must victims suspect their victimization was a hate crime, but they must also have at least one of three criteria met to be classified as a hate crime: the offender used hate language, the offender used hate symbols, or the police told the victim that the crime was a hate crime. Meaning, some victims may have considered their victimization a hate crime that was not ultimately classified as such. Here, we repeat our analysis based on victims who suspected their victimization was a hate crime even if it was not classified as such. We tend to find patterns consistent with results reported in the main text. There are more differences in the bias-motivation, victim's income, the relationship between the victim and offender, and emotional and physical responses to victimization between suspected and BJS-defined hate crimes.

Results

Rates and types of suspected hate crime victimization by SOGI

The violent suspected hate crime victimization rate for LGBT people was 17.7 (SE = 6.6) victimizations per 1,000 LGBT persons compared to 1.6 (SE = 0.2) victimizations per 1,000 non-LGBT persons (OR = 11.4, CI = 2.78, 20.05). The violent sexual orientation- or gender-based suspected hate crime victimization rate for LGBT people was 15.8 (SE = 6.5) victimizations per 1,000 LGBT people compared to 0.7 (SE = 0.2) victimizations per 1,000 non-LGBT people (OR = 24.6, CI = 2.70, 46.47).

About 20.9% (SE = 4.7) of all violent victimizations against LGBT victims were suspected hate crimes compared to 7.8% (SE = 0.7) of all violent victimizations with non-LGBT victims (OR = 3.12, CI = 1.76, 5.54). Relative to non-LGBT people, LGBT people had higher odds of being victims of a violent suspected hate crime (OR = 7.83, CI = 5.42, 11.31).

Of violent suspected hate crimes, 69.9% (SE = 11.8) against LGBT victims were simple assaults compared to 62.8% (SE = 4.6) against non-LGBT victims (OR = 1.38, CI = 0.41, 4.59). Among serious violent crimes, 19.7% (SE = 9.6) against LGBT victims were robberies compared to 6.5% (SE = 1.7) against non-LGBT victims (OR = 3.50, CI = 0.88, 13.96), and 5.4% (SE = 3.0) against LGBT victims were aggravated assaults compared to 20.6% (SE = 3.6) against non-LGBT victims (OR = 0.22, CI = 0.06, 0.75).

The types of bias that motivated violent suspected hate crimes against LGBT and non-LGBT victims were documented in Table S1. Overall, 89.5% of LGBT suspected hate crime victims compared to 38.3% of non-LGBT suspected hate crime victims identified gender or sexuality as the bias motivation.

Table S1. The type of bias-motivation among suspected hate crime victims, by sexual orientation and gender identity, United States, National Crime Victimization Survey 2017-2019.

	**LGBT**		**Non-LGBT**		**OR**	**(95% CI)**
	%	(SE)	%	(SE)	(ref. Non-LGBT)	
**Race**	9.0	(3.8)	72.0	(4.2)	**0.04**	**(0.01, 0.10)**
**Religion**	15.6	(10.6)	11.9	(2.6)	1.37	(0.25, 7.61)
**Ethnicity**	5.5	(2.8)	31.0	(4.7)	**0.13**	**(0.04, 0.40)**
**Disability**	22.6	(12.8)	10.3	(2.5)	2.54	(0.52, 12.38)
**Gender**	66.4	(12.8)	37.4	(5.4)	**3.31**	**(1.01, 10.87)**
**Sexuality**	38.2	(13.9)	4.5	(1.8)	**13.18**	**(3.17, 54.81)**
**Association[a]**	9.5	(4.0)	18.7	(4.1)	0.46	(0.16, 1.32)
**Perceived characteristics[b]**	4.1	(2.4)	8.2	(2.0)	0.47	(0.13, 1.77)
**Gender or sexuality (combined)**	89.5	(4.6)	38.3	(5.3)	**13.64**	**(4.82, 38.61)**

Victims could select more than one bias motivation that apply to their victimization. Bold odds ratios and confidence intervals are statistically significant at $p < .05$.

[a]Association refers to the victim's perception of victimization was motivated by the victim's association with people who have certain characteristics or religious beliefs.

[b]Perceived characteristics refers to the victim's perception of victimization was motivated by the offender's perception of the victim's characteristics or religious beliefs even if inaccurate.

Characteristics of violent suspected hate crime victimization

Table S2 documented whether LGBT and non-LGBT hate crime victims differ in their characteristics, the location of the hate crime victimization, victim-offender relationship, and offender characteristics.

Table S2. Victimization characteristics of violent suspected hate crime, LGBT versus non-LGBT, United States, National Crime Victimization Survey 2017–2019.

	**LGBT**		**Non-LGBT**		**Difference**		**OR**	**(95% CI)**
	%	SE	%	SE	%	SE	(ref. Non-LGBT)	
**Victim Characteristics**								
Current Gender								
Man	17.6	6.7	51.5	4.7	-33.9	8.0	0.20	(0.08, 0.53)

Woman	78.0	8.1	48.5	4.7	29.4	9.2		
Non-binary	2.0	1.4	--	--	--	--	--	--
Person of color	23.7	10.4	46.3	5.7	-22.7	12.3	0.36	(0.10, 1.27)
Below 35 years old	63.5	14.4	36.3	4.8	27.2	15.2	3.05	(0.84, 11.12)
Annual income < $25,000	64.8	13.5	25.0	3.3	**39.8**	**14.2**	**5.52**	**(1.58, 19.32)**
**Location of Incident**								
At or near victim’s home	45.8	16.2	29.6	4.3	16.2	16.2	2.01	(0.55, 7.39)
In public space	44.0	17.3	29.1	3.8	14.9	17.7	1.91	(0.46, 8.04)
**Victim-Offender Relationship**								
Well-known	23.4	11.7	13.3	3.5	10.1	12.5	1.99	(0.46, 8.61)
Stranger	61.2	14.7	61.6	1.7	-0.4	16.2	0.98	(0.26, 3.77)
**Urbanicity of Victim’s Residence**								
Urban	53.9	15.8	47.1	5.2	6.8	16.7	1.31	(0.34, 5.00)
Suburban	39.7	14.8	46.8	5.1	-7.1	15.7	0.76	(0.21, 2.82)
Rural	6.5	4.3	6.1	1.6	0.4	4.6	0.97	(0.20, 4.78)
**Offender Characteristics**								
Offender Sex								
Male(s)	76.1	13.5	70.1	4.5	6.0	13.3	1.36	(0.32, 5.72)
Female(s)	12.1	9.7	21.1	3.8	-9.0	10.6	0.52	(0.08, 3.36)
Male(s) and Female(s)	11.8	10.5	8.8	2.9	2.9	10.9	1.38	(0.16, 11.62)
One offender	71.9	13.3	79.6	3.2	-7.7	14.3	0.66	(0.16, 2.75)
White	83.3	9.7	50.2	5.9	**33.1**	**10.1**	**4.94**	**(1.27, 19.19)**
30 years old or older	59.6	15.6	54.6	5.4	5.0	15.9	1.23	(0.33, 4.52)

None of the percentages should sum to 100 except for urbanicity of incident due to excluded categories that are not reported. Bold differences and odds ratios are statistically significant at $p < .05$.

Social, mental, and physical health correlates of suspected hate crimes among LGBT victims

Table S3 compared social, mental, and physical health correlates of violent victimizations among LGBT victims of hate crimes and LGBT non-hate crime victims.

Table S3. Effects of victimization of violent suspected hate and non-hate crime among LGBT victims, United States, National Crime Victimization Survey 2017–2019.

	Violent Suspected Hate Victims		Violent Non-suspected Hate Victims		Difference		OR	(95% CI)
	%	SE	%	SE	%	SE	(ref. Violent non-hate victims)	
**Problems in Social Life**								
Problems in work or school	35.9	14.5	20.9	3.5	15.0	14.0	2.12	(0.63, 7.09)
Problems with family or friends	38.5	14.7	21.2	3.2	17.3	13.8	2.33	(0.74, 7.28)
Sought help from victim agencies other than the police	15.3	10.6	10.5	2.1	4.8	10.4	1.54	(0.31, 7.61)
**Emotional Responses to Victimization**								
Moderately or severely distressing to be a victim	54.1	17.1	51.1	17.1	3.0	14.9	1.13	(0.34, 3.71)
Worried or anxious	59.6	17.8	40.4	4.4	19.2	15.8	2.17	(0.59, 7.99)
Angry	60.1	17.9	41.5	4.6	18.5	15.8	2.12	(0.57, 7.80)
Sad or depressed	53.5	17.0	28.5	3.8	25.1	15.6	2.90	(0.85, 9.89)
Vulnerable	59.1	17.8	35.7	4.7	23.3	16.2	2.59	(0.69, 9.71)
Violated	50.8	16.3	34.6	4.4	16.2	15.0	1.95	(0.60, 6.36)
Mistrust	54.6	17.1	34.8	5.2	19.7	16.0	2.25	(0.63, 8.03)
Unsafe	59.5	17.8	37.8	4.3	21.7	15.9	2.42	(0.65, 8.95)
Sought professional help for emotional problems relating to victimization	41.5	15.2	14.6	2.6	26.9	14.8	**4.14**	**(1.24, 13.83)**
**Physical Responses to Victimization**								
Victimization resulting in injury	16.6	8.2	26.6	4.1	-10.0	8.5	0.55	(0.17, 1.78)
Headaches	44.4	16.0	15.9	4.0	28.5	15.5	**4.24**	**(1.19, 15.11)**
Trouble sleeping	51.5	16.8	28.6	3.7	22.9	15.3	2.65	(0.80, 8.75)
Changes in eating or drinking	48.7	16.4	20.1	3.7	28.6	15.5	**3.77**	**(1.11, 12.77)**
Upset stomach	45.8	16.1	19.5	3.4	26.3	15.1	**3.49**	**(1.06, 11.47)**
Fatigue	49.9	16.5	25.2	2.9	24.7	15.2	2.96	(0.91, 9.57)
High blood pressure	24.5	12.8	6.9	1.8	17.6	12.3	**4.38**	**(1.21, 15.77)**
Muscle tension	47.7	16.3	19.6	4.0	28.1	15.5	**3.74**	**(1.09, 12.76)**

Sought professional help for physical problems relating to victimization	29.0	13.5	8.5	1.9	20.5	13.2	**4.39**	**(1.22, 15.76)**

Bold differences and odds ratios are statistically significant at $p < .05$.
